# E3 Ligase TRIM21 Deficiency-Driven GAS2L3 Accumulation Facilitates Oncogenic Progression in Breast Cancer

**DOI:** 10.3390/ijms27219937

**Published:** 2026-11-06

**Authors:** Peiying Li, Chunyan Zhao, Yuanyuan Ren, Yuannan Wei, Nan Zhang, Lingbiao Xin, Yanan Sun, Jie Yang, Jinyan He, Xin Liu, Xingjie Gao

**Affiliations:** 1 State Key Laboratory of Experimental Hematology, Key Laboratory of Cellular and Molecular Immunology in Tianjin, and Key Laboratory of Immune Microenvironment and Disease (Ministry of Education), The Province and Ministry Co-Sponsored Collaborative Innovation Center for Medical Epigenetics, Key Laboratory of Breast Cancer Prevention and Therapy (Ministry of Education), School of Basic Medical Science, Tianjin Medical University, Tianjin 300070, China; 2 Department of Biochemistry and Molecular Biology, Department of Immunology, School of Basic Medical Science, Tianjin Medical University, Tianjin 300070, China; 3 Department of Immunology, University of Manitoba, Winnipeg, MB R3E 0T5, Canada

**Keywords:** GAS2L3, breast cancer, TRIM21, ubiquitination, E3 ligase, proliferation

## Abstract

GAS2L3, a member of the growth arrest-specific 2 protein family, is abnormally upregulated in multiple human cancers, implying its potential oncogenic role. However, the mechanisms underlying its stabilization and accumulation during breast cancer progression remain unclear. This study aimed to clarify the regulatory mechanism and oncogenic role of GAS2L3 in breast cancer. We analyzed the expression and prognostic value of GAS2L3 and its related signaling pathways using public breast cancer databases, and conducted functional assays to assess the malignant phenotypes of breast cancer cells. Mass spectrometry and co-immunoprecipitation were performed to identify and verify TRIM21 as a binding partner of GAS2L3. Ubiquitination and cycloheximide chase assays confirmed that TRIM21 facilitates the ubiquitination and proteasomal degradation of GAS2L3, while immunohistochemical staining of clinical specimens and mouse tissues verified their negative correlation. Rescue experiments further validated this regulatory axis. High GAS2L3 expression predicted poor prognosis and promoted breast cancer cell proliferation, migration, and invasion. Loss of TRIM21-mediated GAS2L3 degradation results in aberrant GAS2L3 accumulation, and the TRIM21–GAS2L3 axis drives breast cancer malignant progression, providing potential prognostic biomarkers and therapeutic targets for breast cancer.

## 1. Introduction

Breast cancer, the most prevalent malignancy among women globally, is characterized by its intricate pathogenesis and heterogeneous clinical manifestations, which arise from dynamic molecular alterations [1,2]. To gain a comprehensive understanding of the disease’s evolution and to develop more effective therapeutic strategies, recent research has increasingly focused on integrating multi-omics approaches, including genomics, transcriptomics, proteomics, and radiomics [1,2,3,4,5]. These studies have unveiled important insights into clonal dynamics and immune landscape remodeling, thereby informing the development of therapeutic strategies [1,2,3,4,5]. Pathological activation of oncogenic pathways is central to tumor progression. Therefore, it is imperative to systematically identify key oncogenic drivers by thoroughly evaluating gene expression patterns, clinical correlations, protein–protein interaction networks, and functional pathways. Our investigation focuses on growth arrest-specific 2-like protein 3, an understudied oncoprotein that exhibits markedly elevated expression in breast carcinoma tissues and is strongly associated with unfavorable clinical outcomes.

GAS2L3 is a member of the GAS2 (growth arrest-specific 2) protein family [6,7,8,9]. This family includes four proteins: GAS2, GAS2L1 (growth arrest-specific 2-like protein 1), GAS2L2 (growth arrest-specific 2-like protein 2), and GAS2L3 [10], each with distinct cellular functions [7]. The human *GAS2L3* gene is located on chromosome 12q23.1 and encodes a protein composed of 694 amino acids, featuring a CH domain for F-actin binding and a GAS2-associated (GAR) domain for microtubule binding [10]. Experimental studies demonstrate GAS2L3′s involvement in critical biological processes. As a downstream target of the DREAM complex, it participates in cytoplasmic division during mitosis [6]. Developmental models reveal its essential functions: zebrafish embryos with GAS2L3 suppression show cranial and ventricular malformations [8], while knockout mice exhibit perinatal lethality or cardiac abnormalities including ventricular enlargement and contractile dysfunction in survivors [9]. Despite its established roles in normal physiology, GAS2L3’s clinical relevance remains poorly characterized.

In this study, we first analyzed the high-expression characteristics of GAS2L3 and its association with clinical prognosis using available clinical data from breast cancer patient cohorts. Elevated GAS2L3 levels were found to enhance breast cancer cell proliferation and drive migration and invasion via EMT regulation. Notably, TRIM21 mediates GAS2L3’s ubiquitination and degradation. In breast cancer, the low expression of TRIM21 leads to GAS2L3 accumulation, thereby promoting cancer cell migration and underscoring the pivotal role of the TRIM21–GAS2L3 regulatory axis in breast cancer progression.

## 2. Results

### 2.1. GAS2L3 Is Highly Expressed in Breast Cancer Tissues and Is Associated with Poor Clinical Prognosis of Patients

**Figure 1:**
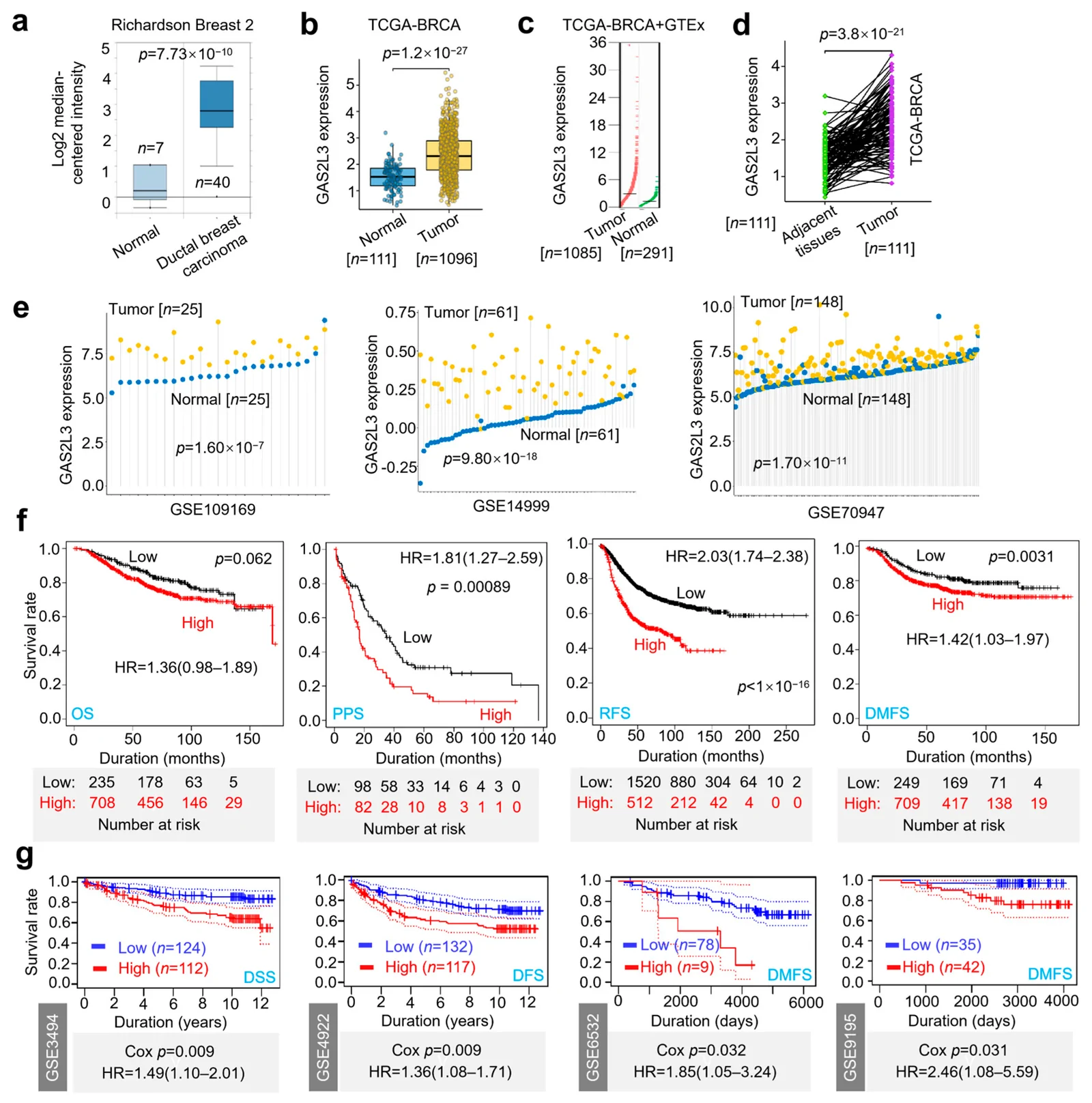
*GAS2L3* is highly expressed and correlated with poor clinical prognosis in breast cancer patients. (**a**) Differential expression of *GAS2L3* mRNA between normal breast tissues (*n* = 7) and ductal breast carcinoma tissues (*n* = 40) based on the Richardson Breast 2 dataset. (**b**,**c**) *GAS2L3* mRNA expression levels in breast cancer tissues relative to normal breast tissues from the TCGA-BRCA or TCGA-BRCA+GTEx datasets. (**d**,**e**) *GAS2L3* mRNA expression differences in breast cancer and paired adjacent paracancerous tissues across four independent datasets, including TCGA-BRCA (*n* = 111), GSE109169 (*n* = 25), GSE14999 (*n* = 61), and GSE70947 (*n* = 148). In panel (**e**), yellow and blue dots represent tumor and normal tissues, respectively. Data visualization was performed using the ggplot2 package in R, and statistical difference analysis was conducted using the Wilcoxon rank-sum test. (**f**) Kaplan–Meier survival analysis of overall survival (OS), post-progression survival (PPS), relapse-free survival (RFS) and distant metastasis-free survival (DMFS) dichotomized by *GAS2L3* mRNA expression in breast cancer patients. (**g**) Cox proportional hazards regression analysis of disease-specific survival (DSS), disease-free survival (DFS) and DMFS based on four independent PrognoScan datasets (GSE3494, GSE4922, GSE6532, and GSE9195) to evaluate the prognostic significance of *GAS2L3*. For panels (**f**) and (**g**), red lines indicate patients with high *GAS2L3* expression, black lines in (**f**) and blue lines in (**g**) indicate patients with low *GAS2L3* expression.

To analyze the mRNA expression profile of *GAS2L3* and its association with breast cancer progression, we first examined multiple clinical transcriptomic datasets. As shown in Figure 1a, *GAS2L3* exhibited significantly higher mRNA expression levels in ductal breast carcinoma tissues (*n* = 40) compared to normal breast tissues (*n* = 7) from the Richardson Breast 2 dataset (*p* = 7.73 × 10^−10^). Consistent with this observation, an analysis of the TCGA-BRCA cohort (Figure 1b, *p* = 1.2 × 10^−27^) and TCGA-BRCA + GTEx dataset (Figure 1c) revealed a higher *GAS2L3* mRNA expression level in breast cancer tissues than in normal controls. Further validation across additional datasets, including TCGA-BRCA (*n* = 111), GSE109169 (*n* = 25), GSE14999 (*n* = 61), and GSE70947 (*n* = 148), demonstrated that *GAS2L3* mRNA expression was significantly upregulated in tumor tissues compared to paired adjacent normal tissues (all *p* < 0.001, Figure 1d,e).

To evaluate whether *GAS2L3* mRNA expression correlates with clinical prognosis in patients with breast cancer, Kaplan–Meier survival analyses were performed using the Kaplan–Meier plotter database. As shown in Figure 1f, although no statistically significant difference in overall survival (OS) was observed between patients with high and low *GAS2L3* expression (*p* = 0.062, HR = 1.36), a trend toward shorter OS was noted in the high-expression cohort. Notably, high *GAS2L3* expression strongly predicted unfavorable post-progression survival (*p* = 0.00089, HR = 1.81), relapse-free survival (*p* < 1 × 10^−16^, HR = 2.03), and distant metastasis-free survival (DMFS, *p* = 0.0031, HR = 1.42) in breast cancer patients. Moreover, Cox proportional hazards regression models were applied to four independent datasets (GSE3494, GSE4922, GSE6532, and GSE9195). As shown in Figure 1g, high *GAS2L3* expression exhibited statistically significant associations with poor disease-specific survival, disease-free survival, and DMFS (all *p* < 0.05, HR > 1). Collectively, these results demonstrate that high *GAS2L3* expression serves as a robust predictor of adverse clinical outcomes in patients with breast cancer.

### 2.2. High GAS2L3 Expression Promotes Breast Cancer Cell Proliferation

**Figure 2:**
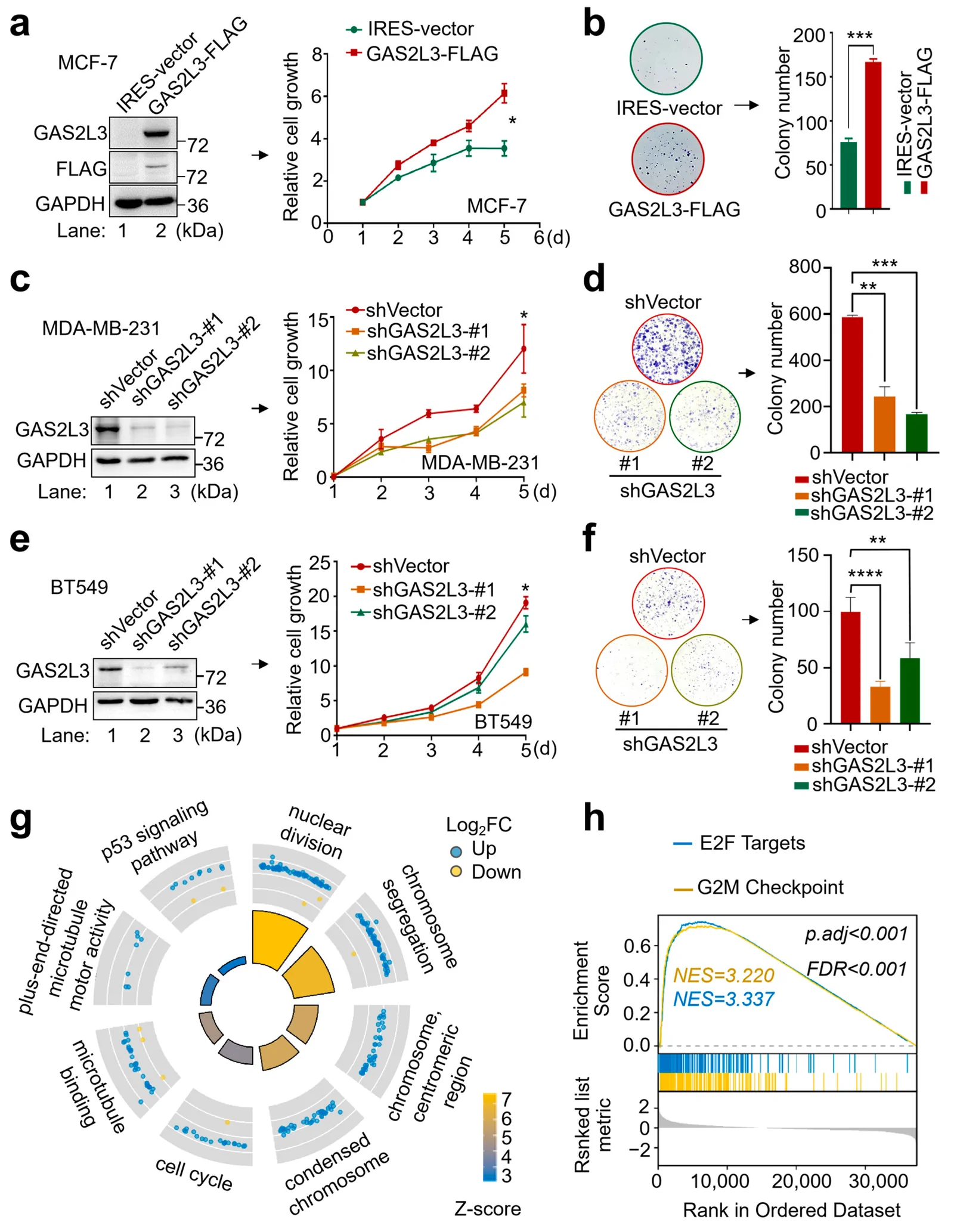
GAS2L3 correlates with cell cycle progression and facilitates breast cancer cell proliferation. (**a**) Western blotting validation of stable GAS2L3-FLAG overexpression in MCF-7 cells. Anti-GAS2L3 and anti-FLAG antibodies were used for protein detection, with GAPDH serving as the internal reference. Cell proliferation was measured via CCK-8 assays. (**b**) Colony formation assays evaluated the colony-forming ability of MCF-7 cells with GAS2L3 overexpression. (**c**–**f**) Western blotting verified the knockdown efficiency of two independent GAS2L3-targeting shRNAs (shGAS2L3-#1 and shGAS2L3-#2) in MDA-MB-231 and BT549 cells. CCK-8 assays (**c**,**e**) and colony formation assays (**d**,**f**) were performed to detect proliferative changes after GAS2L3 silencing. Colony numbers were quantified using ImageJ 1.54g., and statistical differences were analyzed via *t*-test or one-way ANOVA (* *p* < 0.05, ** *p* < 0.01, *** *p* < 0.001, **** *p* < 0.0001). (**g**) The TCGA-BRCA cohort was stratified into high- and low-GAS2L3-expression groups according to the median cutoff. Differentially expressed genes (DEGs) were screened using DESeq2, followed by Gene Ontology (GO) and Kyoto Encyclopedia of Genes and Genomes (KEGG) pathway enrichment analyses with the clusterProfiler package. Enrichment results were visualized as circular plots using the GOplot and ggplot2 packages. (**h**) Gene Set Enrichment Analysis (GSEA) was performed to analyze the enrichment signatures of E2F targets and G2/M checkpoint pathways in cells with high GAS2L3 expression.

To further investigate the role of GAS2L3 in breast cancer progression, we systematically evaluated its effect on cell proliferation in MCF-7, MDA-MB-231, and BT549 cell lines. MCF-7 cells were transfected with a GAS2L3-FLAG overexpression plasmid or the empty IRES-vector. Western blot analysis confirmed efficient GAS2L3 overexpression in the experimental group (Figure 2a). Subsequent Cell Counting Kit-8 (CCK-8) and colony formation assays showed that the ectopic overexpression of GAS2L3 significantly enhanced both cell growth rates and colony-forming capacity (*p* < 0.05) relative to the vector control (Figure 2a,b). To further validate these findings, endogenous GAS2L3 was knocked down in both MDA-MB-231 and BT549 cells using two independent shRNAs, including shGAS2L3-#1 and shGAS2L3-#2. As shown in Figure 2c–f, the knockdown of GAS2L3 resulted in markedly reduced proliferation rates and diminished colony formation (*p* < 0.05), compared to the shVector sample. Notably, the two independent shRNAs exhibited distinct knockdown efficiencies between the two cell lines (Figure 2c,e). Nevertheless, within each cell line, the magnitude of the anti-proliferative and anti-clonogenic effects of GAS2L3 silencing paralleled the knockdown efficiency of the corresponding shRNA, indicating a dose-dependent response to GAS2L3 depletion.

Additionally, transcriptomic analysis of the TCGA-BRCA cohort stratified by *GAS2L3* expression (median cutoff) identified 1794 differentially expressed genes (1240 upregulated, 554 downregulated) (Figure S1a). Gene Ontology (GO) and Kyoto Encyclopedia of Genes and Genomes (KEGG) enrichment analyses highlighted significant associations with cell cycle-related processes, including nuclear division, microtubule binding, and the p53 signaling pathway (Figure 2g and Figure S1b,c). Gene Set Enrichment Analysis (GSEA) also linked high expression of *GAS2L3* to E2F targets (NES = 3.337, FDR < 0.001) and G2/M checkpoint signatures (NES = 3.220, FDR < 0.001) (Figure 2h). We next conducted Spearman correlation analysis to identify genes co-expressed with *GAS2L3*. Representative co-expressed genes, including *REEP6* and *PARPBP*, were shown as heatmap and scatter plots in Figure S2a,b. GO and KEGG analyses indicated these genes were involved in cell cycle events like chromosome segregation and organelle fission (Figure S2c). Overall, these findings suggest that high GAS2L3 expression facilitates breast cancer cell proliferation via modulation of cell cycle progression.

### 2.3. GAS2L3 Promotes Migration and Invasion of Breast Cancer Cells by Regulating the EMT Process

**Figure 3:**
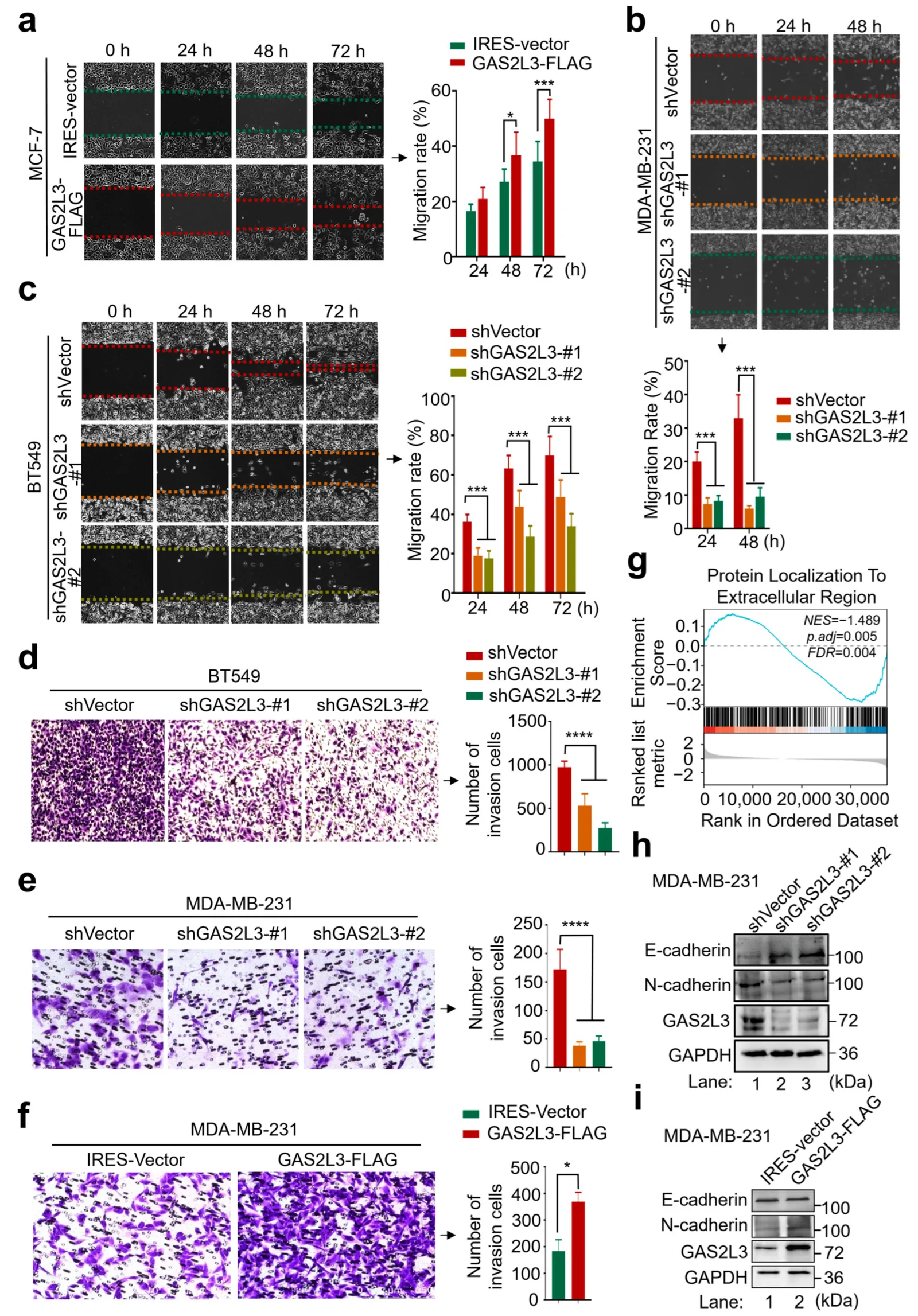
GAS2L3 knockdown inhibits migration, invasion, and EMT progression in breast cancer cells. (**a**) Wound-healing assays were performed in MCF-7 cells with stable GAS2L3-FLAG overexpression or empty vector control. Cell migration was photographed at 0, 24, 48, and 72 h, and migration rates were quantified. (**b,c**) Wound-healing assays were carried out to detect migratory abilities of MDA-MB-231 and BT549 cells with stable GAS2L3 knockdown. (**d**,**e**) Transwell invasion assays were used to evaluate invasive capabilities of GAS2L3-silenced BT549 and MDA-MB-231 cells. (**f**) Transwell invasion assays were conducted in MDA-MB-231 cells with GAS2L3 overexpression. (**g**) GSEA in the TCGA-BRCA cohort stratified by median *GAS2L3* expression (high vs. low subgroups). The enrichment plot is shown for the gene set “protein localization to extracellular region” (NES = −1.489, *p*.adj = 0.005). Genes were ranked by correlation with *GAS2L3* expression; the green curve indicates the running-sum enrichment score profile, and the red–blue heatmap reflects the ranked list metric (red, genes positively correlated with *GAS2L3* expression; blue, genes negatively correlated). (**h**,**i**) Western blotting was used to detect protein levels of GAS2L3 and EMT markers (E-cadherin and N-cadherin), with GAPDH as the loading control, in (**h**) GAS2L3-knockdown and (**i**) GAS2L3-FLAG-overexpressing MDA-MB-231 cells. Statistical differences were analyzed via two-way ANOVA with Sidak’s multiple comparisons test for wound-healing assays (* *p* < 0.05, *** *p* < 0.001) and *t*-test or one-way ANOVA for other experiments (* *p* < 0.05, **** *p* < 0.0001).

We next explored the effect of GAS2L3 expression on the migratory and invasive capabilities of breast cancer cells. Wound-healing assays were first carried out in MCF-7 cells with stable GAS2L3 overexpression. Cell migration was photographed every 24 h, and wound closure rates were quantitatively analyzed. As shown in Figure 3a, the GAS2L3-FLAG group displayed significantly faster wound healing than the empty IRES-vector group, particularly at 72 h (*p* < 0.001), suggesting enhanced migration. Consistent results were obtained in GAS2L3-silenced MDA-MB-231 and BT549 cells. Compared with the non-targeting shVector group, both shGAS2L3-#1 and shGAS2L3-#2 groups displayed remarkably delayed wound closure (Figure 3b,c). These findings suggest that GAS2L3 knockdown impairs the migratory ability of breast cancer cells.

Transwell invasion assays were subsequently performed to assess the effect of GAS2L3 on breast cancer cell invasion. Knockdown of endogenous GAS2L3 markedly reduced the number of invaded cells recovered from the lower chamber in BT549 and MDA-MB-231 cells (Figure 3d,e, *p* < 0.0001). Conversely, MDA-MB-231 cells with GAS2L3 overexpression showed elevated invasive capacity relative to vector control cells (Figure 3f).

Considering the critical role of the extracellular matrix (ECM) in epithelial cell adhesion, as well as tumor invasion and distant metastasis [11], we further performed GSEA to examine the association between GAS2L3 expression and extracellular-related gene programs. The gene set “protein localization to extracellular region” was significantly enriched (Figure 3g, NES = −1.489, *p*.adj = 0.005), suggesting an altered extracellular protein composition in tumors with high GAS2L3 expression. We thus hypothesized that GAS2L3 promotes invasion and migration by influencing the epithelial–mesenchymal transition (EMT) process. We detected the protein levels of canonical EMT markers, specifically E-cadherin and N-cadherin, and the results demonstrated that GAS2L3 knockdown upregulated E-cadherin expression and downregulated N-cadherin expression (Figure 3h), whereas GAS2L3-FLAG overexpression produced the opposite changes in MDA-MB-231 cells (Figure 3i). These changes indicated that GAS2L3 promotes EMT progression in breast cancer cells. In summary, elevated GAS2L3 accelerates breast cancer migration and invasion, and this oncogenic effect is dependent on the activation of the EMT program.

### 2.4. Protein Interaction Network and Cluster Analysis of GAS2L3 in Breast Cancer Cells

**Figure 4:**
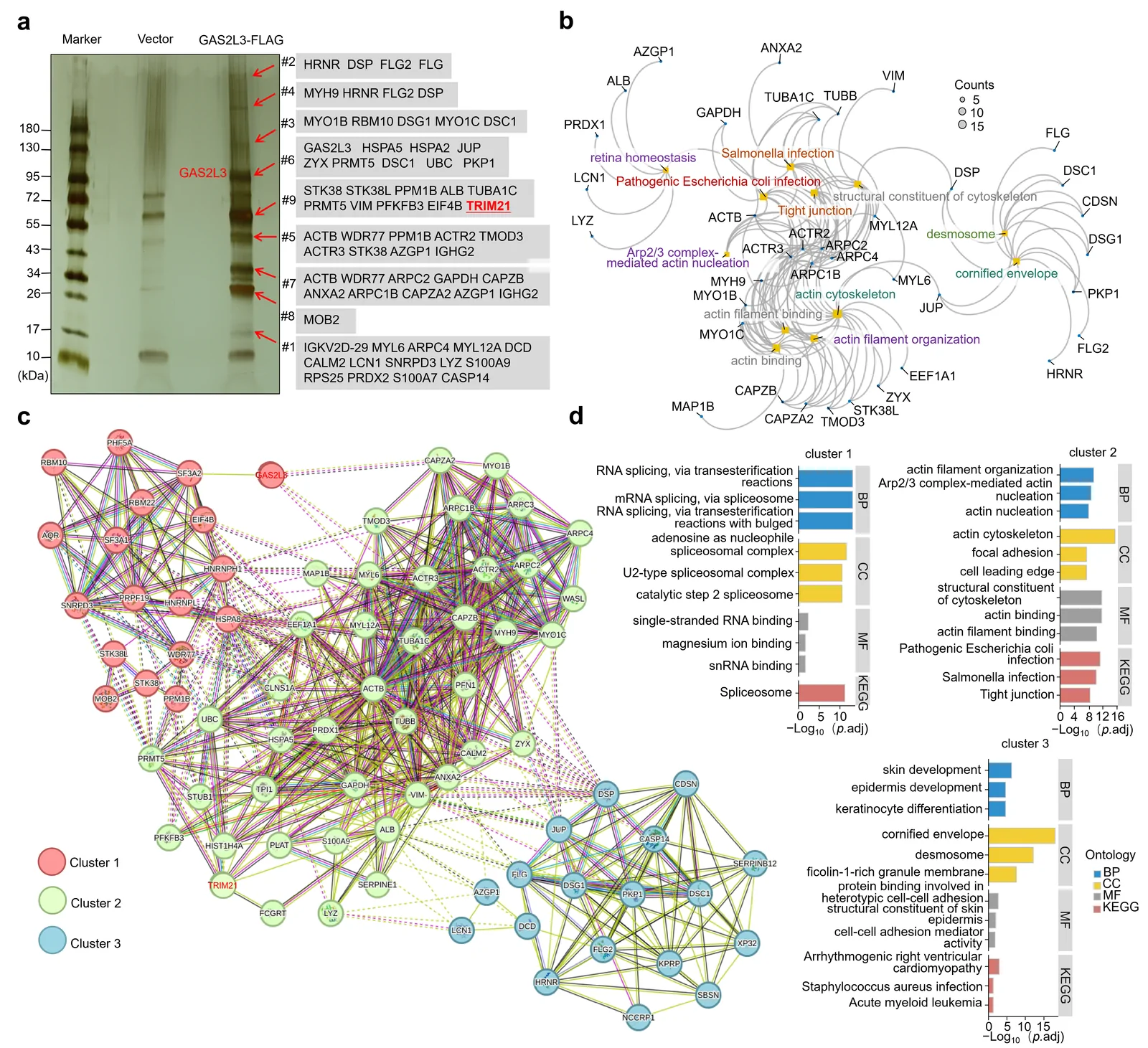
Mass spectrometry identification and clustering analysis of GAS2L3-interacting proteins in breast cancer cells. (**a**) Silver staining following electrophoretic separation of proteins pulled down from MDA-MB-231 cells with stable GAS2L3-FLAG overexpression and empty vector control cells. Red arrows indicate the differential bands (#1–#9) excised for mass spectrometry; the proteins identified in each band are listed in the corresponding boxes on the right. Distinct protein bands were excised and subjected to mass spectrometry to screen potential GAS2L3-interacting proteins. (**b**) GO and KEGG pathway enrichment analyses of all candidate GAS2L3 binding proteins identified by mass spectrometry. (**c**,**d**) A protein–protein interaction network was constructed using the STRING database, followed by K-means clustering analysis. All interacting proteins were classified into three independent clusters: cluster 1 (*n* = 18), cluster 2 (*n* = 42), and cluster 3 (*n* = 18). GO and KEGG enrichment analyses were further performed for proteins in each cluster, and the results are shown as bar graphs.

To elucidate the potential molecular mechanisms by which GAS2L3 drives breast cancer progression, we performed immunoprecipitation combined with mass spectrometry (IP-MS) to identify proteins that interact with GAS2L3. Total protein lysates were extracted from MDA-MB-231 cells with stable GAS2L3-FLAG overexpression and vector control cells. Anti-FLAG antibody-based immunoprecipitation was conducted to capture GAS2L3 and its endogenous binding partners. Following electrophoretic separation and silver staining, we observed distinct differential protein bands between the two groups. Specific bands (#1–#9) were excised and analyzed by mass spectrometry. As shown in Figure 4a, we confirmed that GAS2L3 interacted with multiple candidate proteins, including TRIM21, ANXA2, and STK38.

We next carried out GO and KEGG enrichment analyses on GAS2L3-interacting proteins to clarify their potential biological functions (Figure 4b). Enrichment results indicated that these interactors were primarily involved in pathogen infection and cellular structural organization. Notably, several cytoskeleton-related functional terms, such as actin filament binding, actin cytoskeleton organization and Arp2/3 complex-mediated actin nucleation, were closely correlated with cell cycle progression described above, further supporting the regulatory effect of GAS2L3 on breast cancer cell cycle. Other enriched items including tight junctions, desmosomes and retina homeostasis reflected additional potential biological functions of GAS2L3.

Furthermore, we constructed a global protein–protein interaction network of all GAS2L3-interacting proteins via the STRING database and further performed K-means clustering to classify functional modules (Figure 4c,d). We finally obtained three independent functional clusters with distinct biological properties. Cluster 1 (*n* = 18) was predominantly associated with RNA splicing events, particularly transesterification reactions and spliceosome assembly. Cluster 2 (*n* = 42) contained proteins functioning in cytoskeletal dynamics and pathogen infection pathways, covering core actin-based processes consistent with our earlier cell cycle findings in Figure 2. Cluster 3 (*n* = 18) centered on epidermal development, cell adhesion and cellular stress response processes. Its major enriched items covered epidermal differentiation, desmosome-mediated adhesion, and several disease-associated KEGG signaling pathways. Collectively, these findings reveal the complex interaction network and diverse biological functions of GAS2L3 in breast cancer cells.

### 2.5. TRIM21 Mediates the Ubiquitination and Degradation of GAS2L3 in Breast Cancer Cells

**Figure 5:**
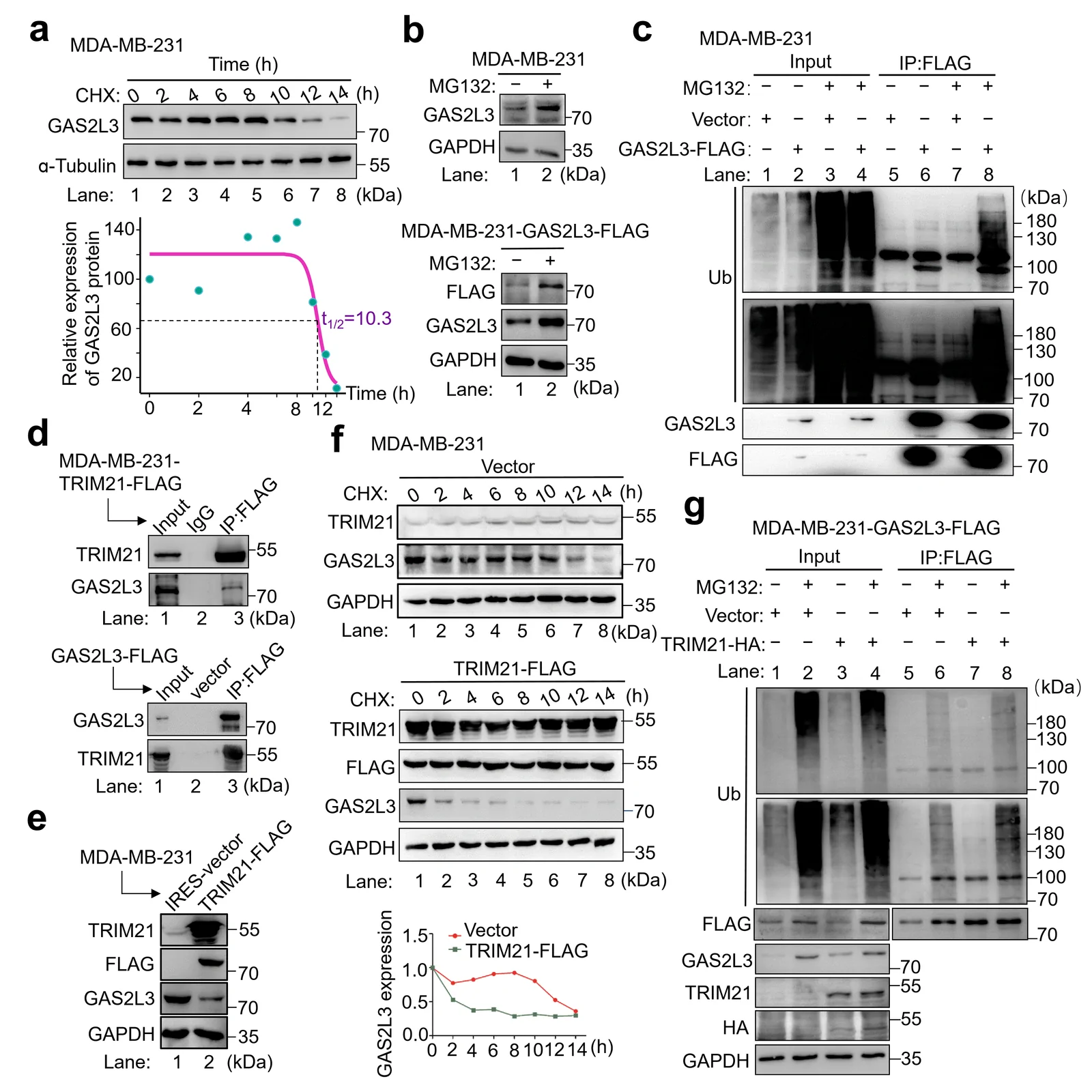
The E3 ubiquitin ligase TRIM21 mediates GAS2L3 ubiquitination in breast cancer cells. (**a**) Western blotting detection of endogenous GAS2L3 protein levels in MDA-MB-231 cells treated with cycloheximide (CHX) for the indicated times (0, 2, 4, 6, 8, 10, 12, 14 h). The protein degradation curve was plotted to calculate the half-life of GAS2L3. Dots indicate relative GAS2L3 protein levels normalized to α-tubulin; the solid line indicates the fitted degradation curve; dashed lines mark the half-life (t_1_/_2_ = 10.3 h). (**b**) Western blotting analysis of endogenous GAS2L3 and exogenous GAS2L3-FLAG protein levels in MDA-MB-231 cells with or without 12-h MG132 treatment. (**c**) GAS2L3-FLAG protein was immunoprecipitated using anti-FLAG antibodies. GAS2L3 ubiquitination levels were detected by anti-ubiquitin (Ub) immunoblotting, with empty vector as the control. Cells were pretreated with MG132 or vehicle prior to harvest. (**d**) Reciprocal co-immunoprecipitation assays verified the binding between TRIM21 and GAS2L3. MDA-MB-231 cells stably expressing either TRIM21-FLAG or GAS2L3-FLAG were used. Anti-FLAG immunoprecipitation of TRIM21-FLAG (upper panel) revealed endogenous GAS2L3 in the precipitate; conversely, anti-FLAG immunoprecipitation of GAS2L3-FLAG (lower panel) revealed endogenous TRIM21. (**e**) Western blotting was carried out to examine protein expression in MDA-MB-231 cells transfected with TRIM21-FLAG or empty vector, using antibodies against TRIM21, FLAG, GAS2L3, and GAPDH. (**f**) CHX chase assays were conducted to compare endogenous GAS2L3 degradation rates with or without TRIM21 overexpression in MDA-MB-231 cells. (**g**) MDA-MB-231 cells stably expressing GAS2L3-FLAG were transfected with TRIM21-HA plasmid or empty vector. After MG132 pretreatment, GAS2L3-FLAG was immunoprecipitated using anti-FLAG antibodies, and GAS2L3 ubiquitination was assessed by anti-ubiquitin immunoblotting.

Among the GAS2L3-interacting proteins identified by IP-MS, we noted TRIM21 protein, a well-documented E3 ubiquitin ligase [12]. As E3 ligases mediate substrate ubiquitination to trigger subsequent proteasomal degradation, we proposed that TRIM21 could destabilize GAS2L3 by catalyzing its ubiquitination, thus initiating downstream proteasomal degradation. To validate this hypothesis, we performed sequential biochemical experiments. We first treated MDA-MB-231 cells with cycloheximide (CHX) to block new protein synthesis and monitor GAS2L3 turnover. The calculated half-life of GAS2L3 was found to be 10.3 h (Figure 5a). We then treated MDA-MB-231 and MDA-MB-231-GAS2L3-FLAG cells with the proteasome inhibitor MG132, and observed obvious accumulation of both endogenous GAS2L3 and GAS2L3-FLAG, indicating that GAS2L3 is degraded via the proteasome pathway (Figure 5b). To directly assess ubiquitination of GAS2L3, we employed an anti-FLAG antibody to selectively isolate the GAS2L3-FLAG fusion protein and detected ubiquitination modification using an anti-Ub antibody. As presented in Figure 5c, robust ubiquitination signals were detected for immunoprecipitated GAS2L3-FLAG after MG132 treatment (lane 8). Taken together, these results confirm that GAS2L3 is modified by ubiquitination and degraded through the proteasome pathway.

To further investigate TRIM21’s role as an E3 ubiquitin ligase for GAS2L3 regulation, we first confirmed the interaction between TRIM21 and GAS2L3 protein by co-immunoprecipitation assays (Figure 5d). Overexpression of TRIM21-FLAG in MDA-MB-231 cells markedly reduced GAS2L3 protein levels compared with the vector control (Figure 5e). We then performed another CHX chase assay in MDA-MB-231 cells with or without TRIM21-FLAG overexpression to compare the intrinsic degradation kinetics of GAS2L3. As shown in Figure 5f, the overexpression of TRIM21-FLAG significantly accelerated the degradation rate of the GAS2L3 protein.

Furthermore, we transfected TRIM21-HA plasmids into MDA-MB-231 cells stably expressing GAS2L3-FLAG to evaluate whether TRIM21 modulates the ubiquitination level of GAS2L3. The GAS2L3-FLAG protein was immunoprecipitated with anti-FLAG antibody, and its ubiquitination level was subsequently examined using anti-ubiquitin antibody. Consistently, TRIM21 overexpression evidently elevated the ubiquitination level of GAS2L3 (lane 8) compared with the vector control group (lane 6) (Figure 5g). Collectively, these results suggest that TRIM21 negatively modulates GAS2L3 protein stability by promoting its ubiquitination, thereby marking GAS2L3 for downstream proteasomal degradation in breast cancer cells.

### 2.6. Absence of TRIM21 Correlates with Accumulation of GAS2L3 in Breast Cancer

**Figure 6:**
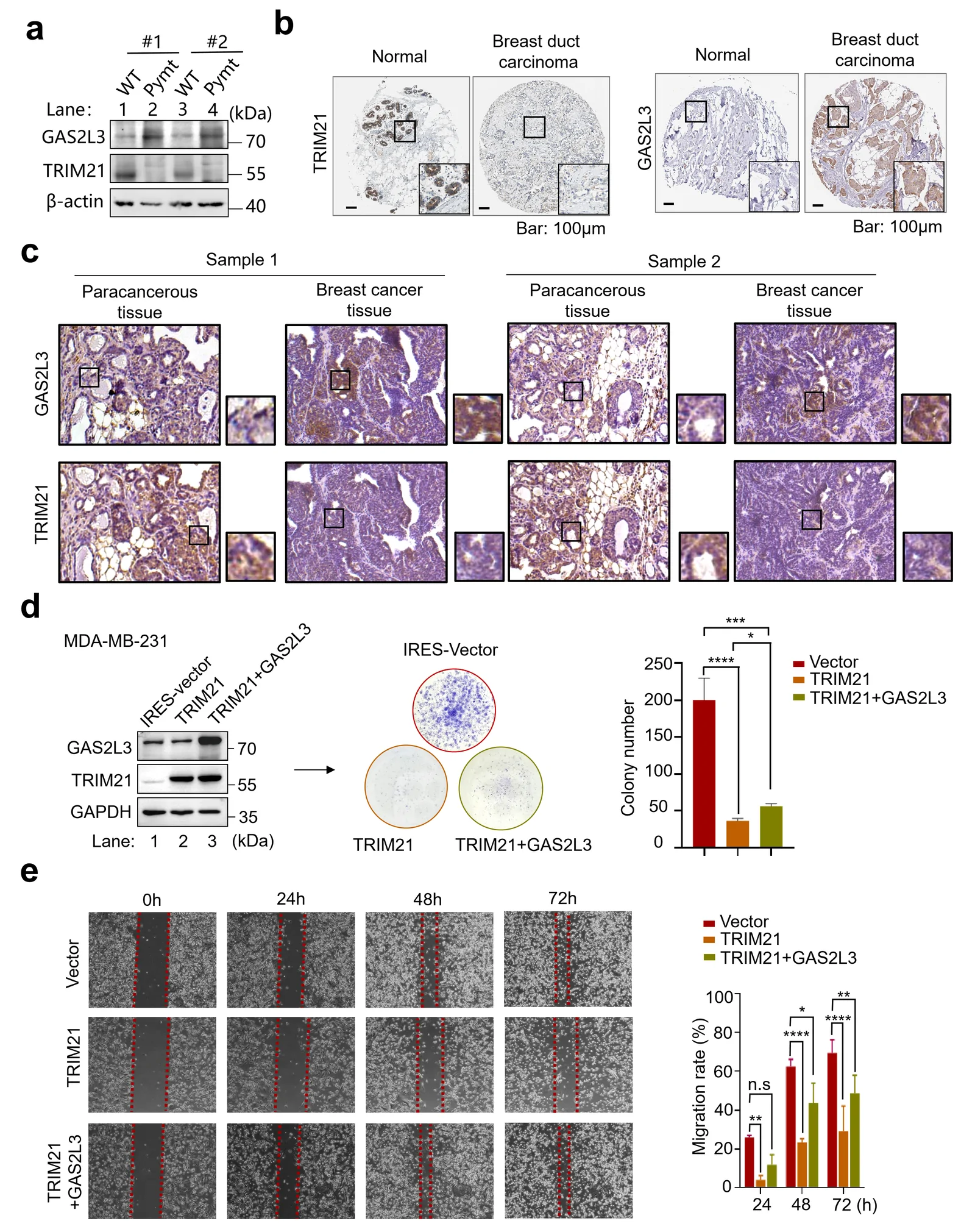
TRIM21 deficiency leads to GAS2L3 accumulation in breast cancer. (**a**) Western blotting analysis of GAS2L3 and TRIM21 protein levels in mammary tissues from wild-type (WT) and MMTV-PyMT mice. β-actin served as the loading control. (**b**) Immunohistochemical (IHC) staining of TRIM21 and GAS2L3 in normal breast tissues and breast ductal carcinoma tissues obtained from the Human Protein Atlas (HPA) database. Scale bar: 100 µm. (**c**) IHC staining of GAS2L3 and TRIM21 in breast cancer and paired paracancerous tissues from PyMT mice. Scale bar: 100 µm. (**d**,**e**) Functional assays were performed in three groups of MDA-MB-231 cells: empty vector control cells, TRIM21-FLAG-overexpressing cells, and TRIM21-FLAG cells with GAS2L3 re-expression. (**d**) Western blotting confirmed TRIM21 and GAS2L3 expression in the three stable cell lines, and colony formation assays were subsequently performed; representative images are shown, and the number of colonies was quantified. (**e**) Wound-healing assays; cell images were captured at 0, 24, 48, and 72 h after scratching, and migration rates were calculated. Statistical analysis was conducted via two-way ANOVA with Sidak’s multiple comparisons test (* *p* < 0.05, ** *p* < 0.01, *** *p* < 0.001, **** *p* < 0.0001).

Given the regulatory role of TRIM21 in GAS2L3 stability, we assessed their expression patterns in mammary tissues from MMTV-PyMT transgenic mice and wild-type (WT) mice. Western blot analysis revealed that TRIM21 was markedly downregulated, whereas GAS2L3 was significantly elevated in PyMT tumors compared with normal mammary glands (Figure 6a). This result suggests an inverse correlation between TRIM21 and GAS2L3 *in vivo*. We then validated this reciprocal pattern in human breast cancer specimens. Analysis of the Human Protein Atlas (HPA) database showed that TRIM21 protein levels were substantially reduced in breast carcinoma tissues relative to normal mammary epithelium, whereas GAS2L3 expression was markedly increased (Figure 6b). Immunohistochemical (IHC) staining further confirmed this opposing distribution in PyMT tumor tissues and matched paracancerous tissues: GAS2L3 was highly expressed within tumor lesions but weakly expressed in adjacent non-tumor regions, whereas TRIM21 exhibited the reverse distribution (Figure 6c).

To further determine the functional relevance of the TRIM21–GAS2L3 axis to breast cancer progression, we generated three stable MDA-MB-231 cell lines expressing empty vector, TRIM21-FLAG, or TRIM21-FLAG with concurrent restoration of GAS2L3 (rescue model). Western blotting was performed to confirm the efficient overexpression of TRIM21 and successful restoration of GAS2L3 in the corresponding cell lines (Figure 6d). Subsequent colony formation assays showed that TRIM21 overexpression markedly reduced the colony-forming capacity of MDA-MB-231 cells, whereas GAS2L3 restoration partially rescued this reduction (Figure 6d). Consistently, wound-healing assays revealed that TRIM21 overexpression significantly suppressed cell migration, and this inhibitory phenotype was also partially rescued following GAS2L3 restoration (Figure 6e). These results indicate that TRIM21 restrains both the clonogenic and migratory capacities of breast cancer cells through destabilization of GAS2L3, as restoring GAS2L3 partially reversed these changes.

GAS2L3 has also been reported to be degraded by the APC/C^Cdh1^ complex through a D-box degron during late mitosis and G1 [13]. We therefore asked whether the accumulation of GAS2L3 in breast cancer might also involve altered activity of this complex. We then analyzed the mRNA expression of *FZR1*, which encodes the APC/C co-activator Cdh1, across multiple breast cancer cohorts. *FZR1* mRNA was significantly upregulated in tumor tissues in the TCGA-BRCA cohort (unpaired, *p* = 3.9 × 10^−11^, Figure S3a; paired, *p* = 2.1 × 10^−4^, Figure S3b), as well as in the GSE109169 (*p* = 7.5 × 10^−5^) and in GSE70947 (*p* = 3.15 × 10^−12^) datasets (Figure S3c). Consistent with the transcriptomic data, IHC staining in the HPA database revealed stronger FZR1/Cdh1 signals in breast ductal carcinoma than in normal mammary epithelium (Figure S3d). As impaired Cdh1 activity may alternatively affect GAS2L3 abundance through cell-cycle redistribution, we further compared the cell-cycle profiles of the rescue groups by flow cytometry. Compared with the vector control, TRIM21-overexpressing cells showed a trend toward an increased G1 fraction, whereas restoration of GAS2L3 reduced the G1 fraction and increased the G2/M fraction; however, none of these changes reached statistical significance (*p* > 0.05, Figure S4a). In parallel, α-tubulin immunofluorescence was performed to examine midbody formation, a marker of cytokinesis progression (Figure S4b). A higher frequency of midbody-positive cells was observed in TRIM21-overexpressing cells, whereas restoration of GAS2L3 reduced the midbody frequency; similarly, this change did not reach statistical significance (*p* > 0.05) but paralleled the direction of the cell-cycle distribution results (Figure S4a). These observations, together with the biochemical and rescue data above, argue against APC/C^Cdh1^ dysregulation as the primary cause of GAS2L3 accumulation and are consistent with TRIM21-mediated degradation as the underlying mechanism in breast cancer. Taken together, our findings support a model in which loss of TRIM21-mediated degradation, rather than reduced APC/C^Cdh1^ activity, accounts for GAS2L3 accumulation and the associated migratory phenotype in breast cancer.

## 3. Discussion

While experimental evidence has reported the physiological role of GAS2L3 in normal organismal development [8,9], its mechanistic contributions to cancer pathogenesis remain incompletely defined. Herein, we validate that GAS2L3 is significantly upregulated in breast cancer, and its overexpression is closely correlated with unfavorable clinical prognosis. We further establish novel functional connections between elevated GAS2L3 expression and enhanced proliferative, invasive, and migratory capacities in breast cancer cells. Notably, this oncogenic association extends beyond breast cancer, with parallel observations of GAS2L3 overexpression correlating with poor prognosis in hepatocellular carcinoma [14] and glioma [15,16], indicating a conserved oncogenic role of GAS2L3 across multiple human cancers. Nevertheless, the molecular determinants driving GAS2L3 accumulation across these malignancies remain elusive. As one of the most vital and evolutionarily conserved post-translational modifications, ubiquitination-mediated proteasomal degradation tightly controls intracellular protein homeostasis and function [17,18]. In this context, our investigation provides the first E3 ligase-centric explanation for GAS2L3 accumulation in breast cancer pathogenesis.

The complete ubiquitination reaction relies on coordinated enzymatic reactions: E1 ubiquitin-activating enzymes prime ubiquitin for transfer, E2 ubiquitin-conjugating enzymes facilitate its covalent attachment to substrates, and E3 ubiquitin ligases confer substrate specificity through the formation of isopeptide bonds [19,20]. Individual substrates can be targeted by multiple E3 ligases, enabling dynamic responses to diverse cellular stimuli [17,18,20,21]. Prior research has identified GAS2L3 as a substrate of APC/C^Cdh1^, which targets its D-box degron to mediate ubiquitin-dependent proteolysis from late mitosis through G1 [13]. Notably, that study examined GAS2L3 regulation in cell-cycle-based systems, and the degradation window of APC/C^Cdh1^ operates within the cell-cycle machinery, whereas the abundance of cell-cycle regulators in tumors reflects a distinct dimension—namely, the overall protein level in tumor tissues relative to normal tissues—rather than their fluctuation across cell-cycle phases. These two dimensions are not mutually exclusive, as exemplified by CDK1, whose activity peaks in mitosis [22] yet whose expression is elevated overall in tumors compared with normal tissues [23]. Consistent with this notion, our cross-cohort analysis showed that FZR1, which encodes Cdh1, is not downregulated at the mRNA level, nor at the protein level, in breast cancer (Figure S3), arguing against impaired APC/C^Cdh1^ activity as the primary cause of GAS2L3 accumulation in tumors. We therefore speculate that APC/C^Cdh1^ and TRIM21 may represent two parallel routes for GAS2L3 turnover, operating at the level of cell-cycle progression and of global protein homeostasis in tumor cells, respectively, and we do not exclude a regulatory role for APC/C^Cdh1^ during cell-cycle phase transitions in tumor cells. In the current study, mass spectrometry analysis successfully identified TRIM21 as a novel E3 ubiquitin ligase targeting GAS2L3. TRIM21 is well recognized as a key tumor suppressor that promotes ubiquitination and degradation of diverse oncogenic substrates to restrain malignant progression [12,24,25]. Our co-immunoprecipitation results further verified the physical interaction between TRIM21 and GAS2L3, while overexpression studies demonstrated TRIM21-dependent enhancement of GAS2L3 ubiquitination in breast cancer cells—establishing GAS2L3 as a bona fide TRIM21 substrate.

Consistent with its tumor-suppressive function, low TRIM21 expression predicts poor prognosis in breast cancer patients [26,27]. Existing studies have demonstrated that TRIM21 downregulation stabilizes multiple oncoproteins including mutant p53 [26], Snail [28,29], and SET7/9 [30] by impairing ubiquitin-mediated degradation. Our data expand the known regulatory spectrum of TRIM21 and demonstrate that TRIM21 deficiency-induced GAS2L3 accumulation is a novel driver event for breast cancer malignancy. Interestingly, GAS2L3 overexpression is induced by DNA hypomethylation in low-grade gliomas [15], whereas this epigenetic regulation has not been reported in glioblastoma or breast cancer, suggesting tissue-specific regulatory mechanisms underlying GAS2L3 dysregulation. Apart from ubiquitination, CDK1-induced phosphorylation has been reported to regulate GAS2L3 mitotic function [13]. Considering most proteins are modulated by multiple post-translational modifications, the crosstalk between phosphorylation and ubiquitination in regulating GAS2L3 oncogenic activity warrants further exploration. Collectively, identification of the TRIM21–GAS2L3 axis deepens the understanding of breast cancer pathogenesis and provides potential therapeutic targets for clinical intervention.

As a cytoskeleton-associated protein [31,32], GAS2L3 plays essential roles in cytokinesis [7] and embryonic development of neural and cardiovascular systems under physiological conditions [8,9]. This multifunctional protein exhibits dual regulatory capacities in cytoskeletal organization and cell cycle progression, suggesting potential mechanistic convergence between these processes [10]. Building upon these functional attributes, combined with enrichment analysis of associated genes and interacting partners, we propose that GAS2L3 exerts oncogenic effects in breast cancer through coordinated modulation of cytoskeletal dynamics and cell cycle control. Our findings align with previous reports of GAS2L3-driven tumorigenesis in gliomas [10], where analogous mechanisms involving cytoskeletal remodeling and cell cycle dysregulation have been observed.

The extracellular matrix critically regulates epithelial cell adhesion and serves as a key determinant of tumor invasiveness and metastatic potential [11]. Mechanistically, aberrant GAS2L3 upregulation may accelerate breast cancer metastasis by modulating cadherin family proteins to disrupt intercellular adhesion. Furthermore, GAS2L3 maintains genomic stability during normal cell division [6]; however, its sustained accumulation triggers defective cytokinesis and chromosome segregation, further inducing replication stress and genomic instability to facilitate tumor progression. Intriguingly, our pathway enrichment analysis revealed significant correlation between GAS2L3 and p53 signaling pathway, which was supported by the regulatory relationship between GAS2L3 and p53 downstream target Necdin [33]. Furthermore, K-means clustering analysis of the GAS2L3 interactome identified several enriched functional modules, including RNA splicing machinery, pathogen–host interactions, epidermal morphogenesis, and developmental signaling pathways. These computational insights provide novel directions for future research to explore the comprehensive biological functions of GAS2L3 in breast cancer.

Several limitations of the present study should be acknowledged. First, although our biochemical data establish TRIM21 as a bona fide E3 ligase for GAS2L3, a potential contribution of other ubiquitin ligases cannot be fully excluded, particularly APC/C^Cdh1^, which targets GAS2L3 from late mitosis through G1 [13]. Our multi-cohort analyses showed that FZR1 is not downregulated in breast cancer, and no significant changes in cell-cycle distribution were observed among the experimental groups (Figures S3 and S4a). Nevertheless, whether TRIM21- and APC/C^Cdh1^-mediated degradation operate in parallel or in a cell-cycle-dependent manner in breast cancer cells remains to be determined. Second, the downstream mechanism by which accumulated GAS2L3 promotes the malignant phenotypes observed in this study, including enhanced proliferation and EMT-associated migration and invasion, remains to be fully elucidated. The additional cytokinesis and cell-cycle analyses (Figure S4) showed directionally consistent trends but did not reach statistical significance, and thus should be interpreted as supporting rather than principal evidence. Our previous work showed that GAS2L3 scaffolds CHMP4B condensates at the midbody to accelerate abscission in hepatocellular carcinoma cells [34]; whether an analogous mechanism operates in breast cancer cells warrants further investigation. Third, the functional assays were performed in a limited panel of breast cancer cell lines covering the luminal and triple-negative subtypes but lacking HER2-enriched models, together with the MMTV-PyMT mouse model. The generalizability of our findings across molecular subtypes of breast cancer therefore warrants further verification. Finally, the clinical relevance of the TRIM21–GAS2L3 axis, although supported by *in vitro* experiments, public IHC data from the HPA database, and the MMTV-PyMT mouse model, has yet to be confirmed in clinical settings. Validation in independent patient cohorts, particularly at the protein level, together with further dissection of the axis in additional breast cancer subtypes and *in vivo* models, will therefore be required to translate these findings into clinical practice.

## 4. Materials and Methods

### 4.1. Analysis of GAS2L3 Expression Characteristics

We analyzed *GAS2L3* mRNA expression using data from multiple datasets, including Richardson Breast 2 [35], TCGA-BRCA (The Cancer Genome Atlas-Breast Invasive Carcinoma, https://portal.gdc.cancer.gov, accessed on 20 October 2026), GTEx (Genotype-Tissue Expression), GSE109169 (*n* = 25), GSE70947 (*n* = 148), and GSE14999 (*n* = 61). To compare GAS2L3 expression between breast cancer and adjacent normal tissues, we used the Wilcoxon rank-sum test and visualized the results with the ggplot2 and car packages in R. In addition, the mRNA expression levels of *FZR1* (encoding the APC/C activator Cdh1)in breast tumor and normal tissues were analyzed in the TCGA-BRCA, GSE109169, and GSE70947 cohorts using the same statistical strategy.

Immunohistochemical data for GAS2L3 and FZR1 proteins in normal and cancerous breast tissues were obtained from the Human Protein Atlas (HPA) database (https://www.proteinatlas.org, accessed on 20 October 2026). In addition, we performed Western blotting to assess TRIM21 and GAS2L3 protein levels in breast tissues from MMTV-PyMT and wild-type (WT) mice, using β-actin as a loading control. We also conducted immunohistochemical staining of GAS2L3 and TRIM21 in breast cancer and adjacent tissues from the MMTV-PyMT mouse model, as previously reported [36]. The study protocol and animal use were approved by the IACUC of Tianjin Medical University.

### 4.2. Clinical Prognostic Assessment

We utilized the Kaplan–Meier plotter platform (http://kmplot.com/analysis/index.php, accessed on 20 October 2026) to analyze overall survival (OS), post-progression survival (PPS), relapse-free survival (RFS), and distant metastasis-free survival (DMFS) prognosis of breast cancer patients with different expression levels of *GAS2L3*. Kaplan–Meier curves were generated to visualize the results. Additionally, Cox regression analyses of disease-specific survival (DSS), disease-free survival (DFS), and DMFS were performed on four independent breast cancer cohorts (GSE3494, GSE4922, GSE6532, GSE9195) retrieved from the PrognoScan database (http://dna00.bio.kyutech.ac.jp/PrognoScan-cgi/PrognoScan.cgi, accessed on 20 October 2026). Survival curves with corresponding hazard ratios (HRs) and 95% confidence intervals were generated.

### 4.3. GAS2L3 Expression-Related Gene Identification and Pathway Enrichment Analysis

After downloading and preprocessing the TCGA-BRCA clinical and expression data, we performed Spearman correlation analysis to identify genes associated with GAS2L3 expression. The results were visualized using the ggplot2 package, which generated heatmaps and correlation scatter plots.

Additionally, breast cancer patients in the TCGA-BRCA dataset were divided into *GAS2L3*-high and *GAS2L3*-low groups based on the median expression value. Differential expression analysis was conducted using DESeq2, with cutoffs defined as |log2FC| > 1 and adjusted *p* < 0.05, identifying 1794 differentially expressed genes (DEGs). The results were visualized using ggplot2.

Subsequently, we performed Gene Ontology (GO) and Kyoto Encyclopedia of Genes and Genomes (KEGG) enrichment analysis on these *GAS2L3*-related genes and DEGs using the clusterProfiler package in R 4.2.1. Furthermore, Gene Set Enrichment Analysis (GSEA) based on the Molecular Signatures Database (MSigDB) was conducted, with a false discovery rate (FDR) < 0.001. Enrichment results were visualized as bubble plots, chord diagrams, circular plots, and enrichment plots using the GOplot and ggplot2 packages.

### 4.4. CCK-8 and Colony Formation Assays

The human breast cancer MCF-7 cell line was transfected with GAS2L3 overexpression plasmids (GAS2L3-FLAG) or empty IRES-vector as negative control. MDA-MB-231 and BT549 cells were transduced with lentiviral shRNA constructs targeting GAS2L3 (pLKO-shGAS2L3-#1 and pLKO-shGAS2L3-#2) or the shVector control. For the rescue experiments, three stable MDA-MB-231 cell lines were used: empty vector, TRIM21-FLAG, and TRIM21-FLAG with concurrent restoration of GAS2L3. The proliferation rate of breast cancer cells was assessed using the CCK-8 kit (CK04, Dojindo, Kumamoto, Japan), and the cell colony formation assay was conducted following a previously reported protocol [15]. Statistical analysis was conducted using GraphPad Prism 7.0.4 software, employing a *t*-test or ANOVA with Sidak’s multiple comparisons test.

### 4.5. Cell Invasion and Migration Assays

To investigate the impact of GAS2L3 expression on breast cancer cell invasion and migration, wound-healing and Transwell assays were conducted. The MCF-7, MDA-MB-231, and BT549 cells with either overexpression or knockdown of GAS2L3, together with their corresponding control cells, were subjected to a wound-healing assay. Cells were seeded in a 6-well plate, and the following day, three vertical lines were drawn in each well to create scratches. Serum-free medium was added, and images were captured at 0 h. The plates were then returned to the incubator for further culture, as reported previously. Cell migration was observed under an inverted microscope at 24 h, 48 h, 72 h, and other time points, after which images were acquired. Migration distances were measured, and migration rates were calculated. Wound-healing assay was also performed in MDA-MB-231 cells stably expressing IRES-vector or TRIM21-FLAG, and MDA-MB-231-TRIM21-FLAG cells with restored GAS2L3 expression.

For the Transwell invasion experiment, diluted Matrigel gel (356231, Corning, NY, USA) was mixed with pre-cooled serum-free medium and evenly coated onto the upper inserts of Transwell chambers. Dilution ratios of RPMI1640: Matrigel = 40:1 for BT549 cells and DMEM: Matrigel = 30:1 for MDA-MB-231 cells were employed. After gel solidification, 1 × 10^5^ BT549 cells or 7 × 10^4^ MDA-MB-231 cells were seeded in the upper chambers. The lower chambers were filled with complete medium containing 10% serum, and the cells were then incubated for 12–16 h. Subsequently, non-invaded cells in the upper chamber were removed, and invaded cells on the lower surface were fixed with 4% paraformaldehyde, stained with crystal violet, and counted.

### 4.6. Detection of EMT-Related Protein Expression

We performed Western blotting to measure the protein expression levels of GAS2L3 and EMT-related markers. For the knockdown analysis, lysates were obtained from MDA-MB-231 cells stably transduced with GAS2L3-targeting shRNAs (shGAS2L3-#1 and shGAS2L3-#2) or the shVector control. For the overexpression analysis, lysates were obtained from MDA-MB-231 cells stably expressing GAS2L3-FLAG or the empty IRES-vector control. Specific antibodies were used to detect GAS2L3, E-cadherin (an epithelial cell marker), N-cadherin (a mesenchymal cell marker), and GAPDH. The antibodies used were anti-E-cadherin (catalog #3195, Cell Signaling Technology, Danvers, MA, USA), anti-N-Cadherin (ab76011, Abcam, Cambridge, UK), anti-GAS2L3 (NBP2-33532, Novus Biologicals, Centennial, CO, USA), and anti-GAPDH (60004-1-Ig, Proteintech, Rosemont, IL, USA).

### 4.7. Mass Spectrometric Identification and Analysis of GAS2L3-Binding Proteins

The stable MDA-MB-231 cells overexpressing GAS2L3-FLAG and the control cells were lysed with TNEN lysate (50 mM Tris-Cl, 2 mM EDTA, 300 mM NaCl, 0.5% NP-40) containing protease inhibitors. Subsequently, the lysate was co-incubated with anti-FLAG M2 affinity beads (A2220, Sigma-Aldrich, St. Louis, MO, USA) for 3 h. Bound proteins were eluted with a FLAG peptide (GLS-YM-180688, GL Biochem Ltd., Shanghai, China) and concentrated using a 3 kDa Amicon Ultra centrifugal filter (MilliporeSigma, Burlington, MA, USA). Eluted proteins were separated by 8% SDS-PAGE electrophoresis and subjected to silver staining. As previously documented, the target protein bands were excised for mass spectrometry analysis, with the vector serving as a control [37,38]. A protein–protein interaction (PPI) network was constructed using STRING based on the identified GAS2L3-binding proteins. K-means clustering was then performed. GO and KEGG pathway enrichment analyses were conducted on the entire GAS2L3-binding protein set and each cluster using the clusterProfiler R package. Enrichment analysis results were visualized as network diagrams using ggplot2, igraph, and ggraph.

### 4.8. GAS2L3 Ubiquitination Analysis

We conducted a series of experiments to investigate whether GAS2L3 undergoes degradation through the ubiquitination pathway. MDA-MB-231 or MDA-MB-231-GAS2L3-FLAG cells were cultured and treated with cycloheximide (CHX, CA0019, Leagene, Beijing, China) for the indicated time points (0, 2, 4, 6, 8, 10, 12, and 14 h) or with MG132 (S2619, Selleck Chemicals, Houston, TX, USA) for 12 h. Cell lysates were collected and subjected to Western blotting using anti-GAS2L3 and anti-GAPDH and anti-α-Tubulin (ab52866, Abcam, Cambridge, UK) antibodies. The collected data was utilized to construct the protein degradation curve of GAS2L3 and determine the half-life (t_1_/_2_) value. To assess ubiquitination, lysates were incubated with Anti-FLAG M2 Affinity Beads. Subsequently, bound proteins were competitively eluted with FLAG peptides and separated by SDS-PAGE. The gels were then probed with anti-FLAG (F1804, Sigma-Aldrich, St. Louis, MO, USA), anti-GAS2L3, and anti-ubiquitin (catalog #3933, Cell Signaling Technology, Danvers, MA, USA) antibodies to detect GAS2L3 ubiquitination.

The interaction between GAS2L3 and the E3 ligase TRIM21 was subsequently validated by co-immunoprecipitation (Co-IP) experiment, as previously reported [39,40]. Briefly, the coding sequence of TRIM21 (NM_003141.4) was cloned into pLVX-IRES-FLAG and pLVX-IRES-HA to generate IRES-TRIM21-FLAG and IRES-TRIM21-HA expression plasmids, respectively. Stable MDA-MB-231 cells overexpressing TRIM21-FLAG were established and treated with CHX for the indicated time points (0, 2, 4, 6, 8, 10, 12, and 14 h). GAS2L3 protein stability was evaluated by Western blotting, and corresponding degradation curves were plotted accordingly. MDA-MB-231-GAS2L3-FLAG stable cells were cultured and transfected with either TRIM21-HA or the vector plasmid. Afterward, the cells were treated with MG132 for 12 h to obtain cell lysate. GAS2L3-FLAG was immunoprecipitated using Anti-FLAG M2 Affinity Beads, and the effect of TRIM21 overexpression on GAS2L3 ubiquitination was examined by Western blotting with anti-ubiquitin antibody.

### 4.9. Flow Cytometry Analysis

Cell-cycle distribution was analyzed by flow cytometry as previously described [34]. Briefly, MDA-MB-231 cells with the indicated treatments were harvested and fixed in 75% ice-cold ethanol at 4 °C overnight. The fixed cells were subsequently stained with propidium iodide in the presence of RNase A, and DNA content was measured on a BD FACSVerse flow cytometer (BD Biosciences, San Jose, CA, USA). Cell-cycle profiles were processed with FlowJo 10.8.1 software, and the proportions of cells in G1, S, and G2/M phases were determined.

### 4.10. Immunofluorescence Assay

Immunofluorescence assay was performed as previously described [34,41]. Briefly, MDA-MB-231 cells were fixed with 4% paraformaldehyde for 10 min, permeabilized with 0.2% Triton X-100 (T8200, Solarbio, Beijing, China), and incubated with a rabbit monoclonal antibody against α-tubulin (ab52866, Abcam, Cambridge, UK) at 4 °C for 12 h. The cells were then incubated with the corresponding fluorescent secondary antibody (donkey anti-rabbit IgG (H+L), Alexa Fluor™ 488, A21206, Thermo Fisher Scientific, Waltham, MA, USA) for 7 h at 4 °C. Following DAPI mounting (F6057, Sigma-Aldrich, St. Louis, MO, USA), images were captured with a laser confocal microscope (Leica DMi8, Leica Microsystems, Wetzlar, Germany), and midbody-positive cells were quantified.

## 5. Conclusions

In summary, our study identifies a novel TRIM21–GAS2L3 regulatory axis that modulates breast cancer progression. Functional experiments confirmed that aberrant upregulation of GAS2L3 promotes the proliferation, migration and invasion of breast cancer cells. Clinically, elevated GAS2L3 expression is closely correlated with unfavorable prognosis in breast cancer patients. Importantly, we demonstrate that TRIM21 functions as a crucial E3 ubiquitin ligase mediating GAS2L3 ubiquitination and degradation. The absence of TRIM21 in breast cancer impairs this degradation pathway, thus triggering abnormal accumulation of endogenous GAS2L3. Collectively, the TRIM21–GAS2L3 signaling axis serves as a key regulator of breast cancer tumorigenesis and progression, offering a promising prognostic biomarker and therapeutic target for clinical breast cancer treatment.

## Data Availability

The data presented in this study are available within the article and its Supplementary Materials. Further inquiries can be directed to the corresponding author.

## Supplementary Materials

The following supporting information can be downloaded at: https://www.mdpi.com/article/10.3390/ijms27219937/s1.

## Abbreviations

The following abbreviations are used in this manuscript:

GAS2	Growth Arrest-Specific 2
GAS2L3	Growth Arrest-Specific 2-Like Protein 3
EMT	Epithelial-Mesenchymal Transition
CHX	Cycloheximide
Co-IP	Co-immunoprecipitation
IP-MS	Immunoprecipitation combined with mass spectrometry
IHC	Immunohistochemical
CCK-8	Cell Counting Kit-8
HPA	Human Protein Atlas
GO	Gene Ontology
KEGG	Kyoto Encyclopedia of Genes and Genomes
GSEA	Gene Set Enrichment Analysis
MSigDB	Molecular Signatures Database
DEGs	Differentially expressed genes
OS	Overall survival
PPS	Post-Progression Survival
RFS	Relapse-Free Survival
DMFS	Distant Metastasis-Free Survival
DSS	Disease-Specific Survival
DFS	Disease-Free Survival
HR	Hazard Ratio
ECM	Extracellular matrix
WT	Wild-type

## References

[1] Nolan E., Lindeman G.J., Visvader J.E. (2023). Deciphering breast cancer: From biology to the clinic. Cell.

[2] Onkar S.S., Carleton N.M., Lucas P.C., Bruno T.C., Lee A.V., Vignali D.A.A., Oesterreich S. (2023). The Great Immune Escape: Understanding the Divergent Immune Response in Breast Cancer Subtypes. Cancer Discov..

[3] Guo Z., Saw P.E., Jon S. (2024). Non-Invasive Physical Stimulation to Modulate the Tumor Microenvironment: Unveiling a New Frontier in Cancer Therapy. BIO Integr..

[4] Conti A., Duggento A., Indovina I., Guerrisi M., Toschi N. (2021). Radiomics in breast cancer classification and prediction. Semin. Cancer Biol..

[5] Vranic S., Cyprian F.S., Gatalica Z., Palazzo J. (2021). PD-L1 status in breast cancer: Current view and perspectives. Semin. Cancer Biol..

[6] Wolter P., Schmitt K., Fackler M., Kremling H., Probst L., Hauser S., Gruss O.J., Gaubatz S. (2012). GAS2L3, a target gene of the DREAM complex, is required for proper cytokinesis and genomic stability. J. Cell Sci..

[7] Fackler M., Wolter P., Gaubatz S. (2014). The GAR domain of GAS2L3 mediates binding to the chromosomal passenger complex and is required for localization of GAS2L3 to the constriction zone during abscission. FEBS J..

[8] Sharaby Y., Lahmi R., Amar O., Elbaz I., Lerer-Goldshtein T., Weiss A.M., Appelbaum L., Tzur A. (2014). Gas2l3 is essential for brain morphogenesis and development. Dev. Biol..

[9] Gründl M., Engel F.B., Gaubatz S. (2017). GAS2L3: Coordinator of cardiomyocyte cytokinesis?. Cell Cycle.

[10] Zhang N., Zhao C., Zhang X., Cui X., Zhao Y., Yang J., Gao X. (2021). Growth arrest-specific 2 protein family: Structure and function. Cell Prolif..

[11] Park M., Kim D., Ko S., Kim A., Mo K., Yoon H. (2022). Breast Cancer Metastasis: Mechanisms and Therapeutic Implications. Int. J. Mol. Sci..

[12] Chen X., Cao M., Wang P., Chu S., Li M., Hou P., Zheng J., Li Z., Bai J. (2022). The emerging roles of TRIM21 in coordinating cancer metabolism, immunity and cancer treatment. Front. Immunol..

[13] Pe’er T., Lahmi R., Sharaby Y., Chorni E., Noach M., Vecsler M., Zlotorynski E., Steen H., Steen J.A., Tzur A. (2013). Gas2l3, a novel constriction site-associated protein whose regulation is mediated by the APC/C Cdh1 complex. PLoS ONE.

[14] Gao X., Zhao C., Zhang N., Cui X., Ren Y., Su C., Wu S., Yao Z., Yang J. (2021). Genetic expression and mutational profile analysis in different pathologic stages of hepatocellular carcinoma patients. BMC Cancer.

[15] Zhao C., Zhang N., Cui X., Zhang X., Ren Y., Su C., He J., Zhang W., Sun X., Yang J. (2021). Integrative analysis regarding the correlation between GAS2 family genes and human glioma prognosis. Cancer Med..

[16] Zhou Y., Zhang L., Song S., Xu L., Yan Y., Wu H., Tong X., Yan H. (2021). Elevated GAS2L3 Expression Correlates With Poor Prognosis in Patients With Glioma: A Study Based on Bioinformatics and Immunohistochemical Analysis. Front. Genet..

[17] Cai C., Tang Y.D., Zhai J., Zheng C. (2022). The RING finger protein family in health and disease. Signal Transduct. Target Ther..

[18] Popovic D., Vucic D., Dikic I. (2014). Ubiquitination in disease pathogenesis and treatment. Nat. Med..

[19] Cockram P.E., Kist M., Prakash S., Chen S.H., Wertz I.E., Vucic D. (2021). Ubiquitination in the regulation of inflammatory cell death and cancer. Cell Death Differ..

[20] Zheng N., Shabek N. (2017). Ubiquitin Ligases: Structure, Function, and Regulation. Annu. Rev. Biochem..

[21] Mahlokozera T., Patel B., Chen H., Desouza P., Qu X., Mao D.D., Hafez D., Yang W., Taiwo R., Paturu M. (2021). Competitive binding of E3 ligases TRIM26 and WWP2 controls SOX2 in glioblastoma. Nat. Commun..

[22] Guardavaccaro D., Pagano M. (2006). Stabilizers and destabilizers controlling cell cycle oscillators. Mol. Cell.

[23] Liu X., Wu H., Liu Z. (2022). An Integrative Human Pan-Cancer Analysis of Cyclin-Dependent Kinase 1 (CDK1). Cancers.

[24] Alomari M. (2021). TRIM21—A potential novel therapeutic target in cancer. Pharmacol. Res..

[25] Jones E.L., Laidlaw S.M., Dustin L.B. (2021). TRIM21/Ro52—Roles in Innate Immunity and Autoimmune Disease. Front. Immunol..

[26] Liu J., Zhang C., Xu D., Zhang T., Chang C.Y., Wang J., Liu J., Zhang L., Haffty B.G., Zong W.X. (2023). The ubiquitin ligase TRIM21 regulates mutant p53 accumulation and gain of function in cancer. J. Clin. Investig..

[27] Zhou W., Zhang Y., Zhong C., Hu J., Hu H., Zhou D., Cao M. (2018). Decreased expression of TRIM21 indicates unfavorable outcome and promotes cell growth in breast cancer. Cancer Manag. Res..

[28] Jin Y., Zhao X., Zhang Q., Zhang Y., Fu X., Hu X., Wan Y. (2020). Cancer-associated mutation abolishes the impact of TRIM21 on the invasion of breast cancer cells. Int. J. Biol. Macromol..

[29] Jin Y., Zhang Y., Li B., Zhang J., Dong Z., Hu X., Wan Y. (2019). TRIM21 mediates ubiquitination of Snail and modulates epithelial to mesenchymal transition in breast cancer cells. Int. J. Biol. Macromol..

[30] Si W., Zhou J., Zhao Y., Zheng J., Cui L. (2020). SET7/9 promotes multiple malignant processes in breast cancer development via RUNX2 activation and is negatively regulated by TRIM21. Cell Death Dis..

[31] Stroud M.J., Kammerer R.A., Ballestrem C. (2011). Characterization of G2L3 (GAS2-like 3), a new microtubule- and actin-binding protein related to spectraplakins. J. Biol. Chem..

[32] Stroud M.J., Nazgiewicz A., McKenzie E.A., Wang Y., Kammerer R.A., Ballestrem C. (2014). GAS2-like proteins mediate communication between microtubules and actin through interactions with end-binding proteins. J. Cell Sci..

[33] Asai T., Liu Y., Di Giandomenico S., Bae N., Ndiaye-Lobry D., Deblasio A., Menendez S., Antipin Y., Reva B., Wevrick R. (2012). Necdin, a p53 target gene, regulates the quiescence and response to genotoxic stress of hematopoietic stem/progenitor cells. Blood.

[34] Zhao Y., Zheng M., Li P., Zhao C., Chen R., Qian B., Cui X., Ren Y., Su C., Shi L. (2026). IDR-driven LLPS of GAS2L3 scaffolds CHMP4B condensates to accelerate cytokinesis in hepatocellular carcinoma cells. J. Adv. Res..

[35] Richardson A.L., Wang Z.C., De Nicolo A., Lu X., Brown M., Miron A., Liao X., Iglehart J.D., Livingston D.M., Ganesan S. (2006). X chromosomal abnormalities in basal-like human breast cancer. Cancer Cell.

[36] Hu L., Zeng Y., Xin L., Yang J. (2023). SND1, a novel co-activator of HIF1α, promotes tumor initiation in PyMT-induced breast tumor. FEBS J..

[37] Chen Y., Kwon S.W., Kim S.C., Zhao Y. (2005). Integrated approach for manual evaluation of peptides identified by searching protein sequence databases with tandem mass spectra. J. Proteome Res..

[38] Fu X., Zhang C., Meng H., Zhang K., Shi L., Cao C., Wang Y., Su C., Xin L., Ren Y. (2018). Oncoprotein Tudor-SN is a key determinant providing survival advantage under DNA damaging stress. Cell Death Differ..

[39] Gao X., Fu X., Song J., Zhang Y., Cui X., Su C., Ge L., Shao J., Xin L., Saarikettu J. (2015). Poly(A)(+) mRNA-binding protein Tudor-SN regulates stress granules aggregation dynamics. FEBS J..

[40] Gao X., Zhao X., Zhu Y., He J., Shao J., Su C., Zhang Y., Zhang W., Saarikettu J., Silvennoinen O. (2012). Tudor staphylococcal nuclease (Tudor-SN) participates in small ribonucleoprotein (snRNP) assembly via interacting with symmetrically dimethylated Sm proteins. J. Biol. Chem..

[41] Wang Y., Wang X., Cui X., Zhuo Y., Li H., Ha C., Xin L., Ren Y., Zhang W., Sun X. (2020). Oncoprotein SND1 hijacks nascent MHC-I heavy chain to ER-associated degradation, leading to impaired CD8(+) T cell response in tumor. Sci. Adv..

