# Supplementary material for: E3 Ligase TRIM21 Deficiency-Driven GAS2L3 Accumulation Facilitates Oncogenic Progression in Breast Cancer
Source: Int J Mol Sci. 2026 Nov 6;27(21):9937. doi: 10.3390/ijms27219937 (PMC13763800; doi:10.3390/ijms27219937)
Supplement: Supplementary file 1 [file ijms-27-09937-s001.zip › ijms-4600689-supplementary.pdf]

## Supplementary Figures

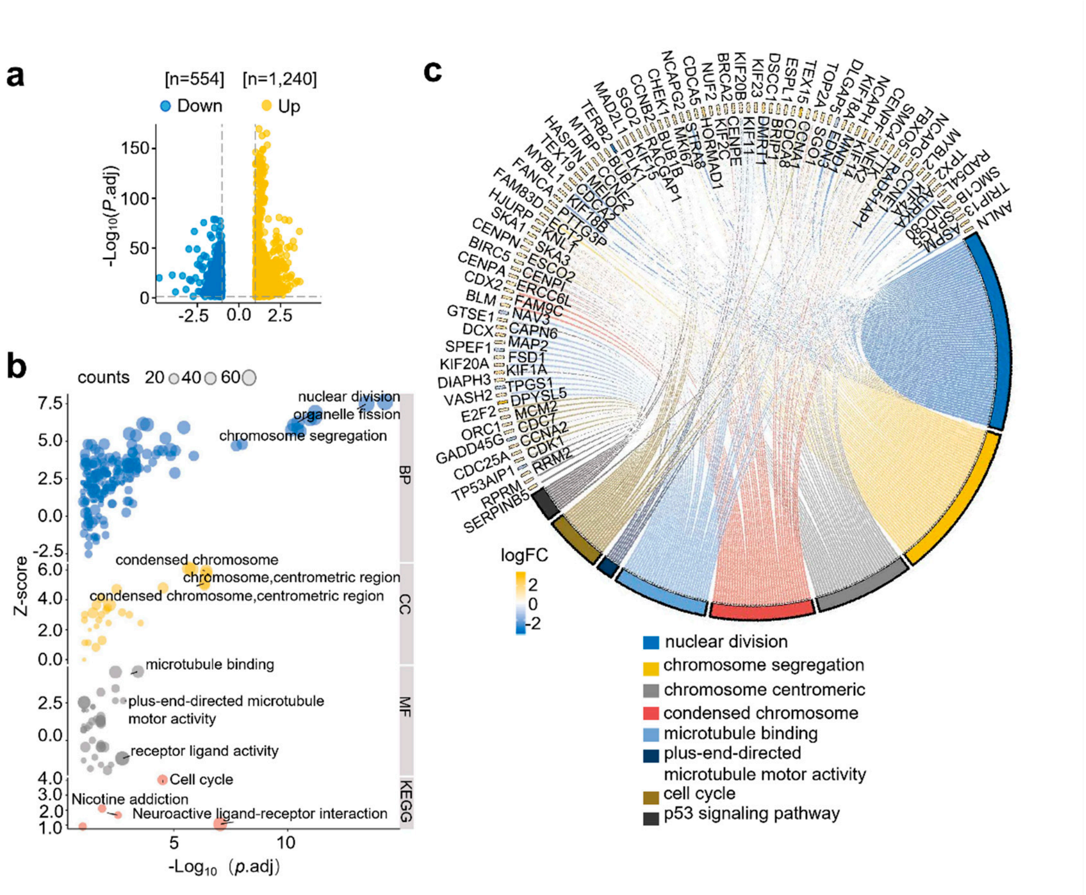


**Figure S1.** Differential gene expression and functional enrichment associated with GAS2L3 in the TCGA-BRCA cohort. The TCGA-BRCA cohort was divided into GAS2L3 high-expression and low-expression subgroups according to the median expression value. Differential expression analysis identified 1,794 DEGs, including 554 downregulated genes and 1,240 upregulated genes, which were displayed in a volcano plot (a). GO and KEGG enrichment analyses were performed to investigate biological functions and signaling pathways enriched in DEGs. Corresponding results were visualized as a bubble plot (b) and a chord diagram (c).

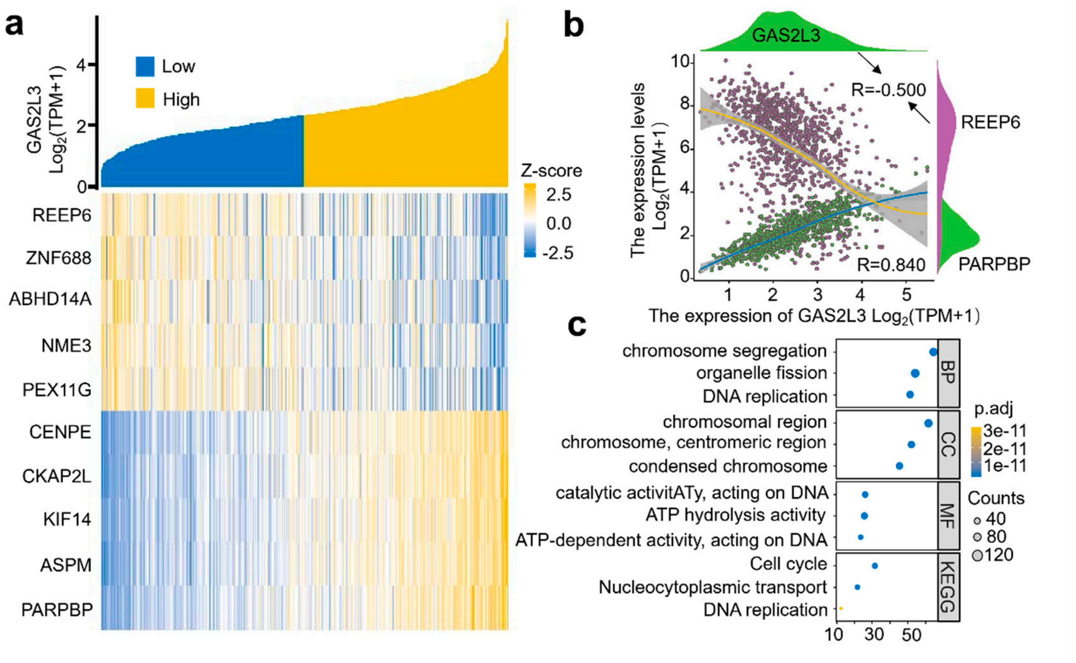


**Figure S2.** Functional enrichment of genes co-expressed with GAS2L3 in breast cancer tissues. (a) Correlation analysis between GAS2L3 and other genes was performed based on expression data from the TCGA-BRCA cohort. Heatmaps showed the top five genes with the strongest positive and negative correlations to GAS2L3. (b) Spearman correlation analysis was applied to verify the correlation between GAS2L3 and two representative co-expressed genes, namely REEP6 (R = -0.500) and PARPBP (R = 0.840). (c) GO and KEGG enrichment analyses were performed on GAS2L3-correlated genes with |R| ≥ 0.4. Enrichment results were visualized as bubble plots.

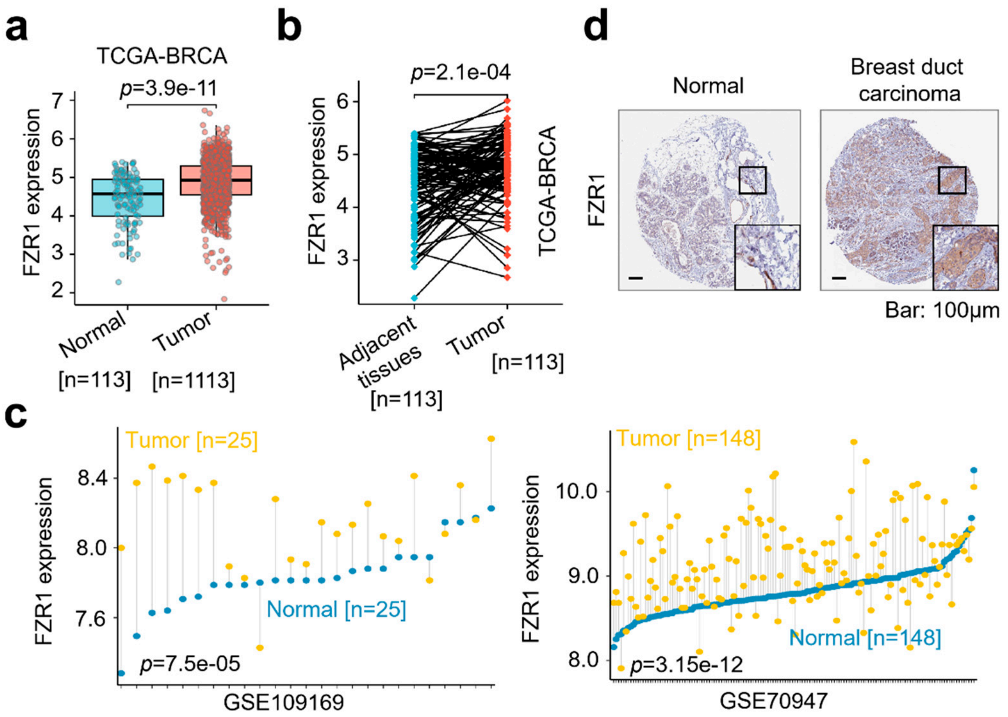


**Figure S3.** Expression of FZR1 in breast cancer patients. (a) Differential expression of FZR1 mRNA between normal breast tissues (n=113) and breast cancer tissues (n=1113) from the TCGA-BRCA datasets. (b, c) FZR1 mRNA expression differences between paired breast cancer and adjacent non-tumor tissues across three independent datasets, including TCGA-BRCA (n=113 pairs), GSE109169 (n=25 pairs), GSE70947 (n=148 pairs). Data visualization was performed using the ggplot2 package in R, and statistical differences were analyzed using the Wilcoxon rank-sum test. (d) Immunohistochemical (IHC) staining for FZR1 in normal breast tissues and breast ductal carcinoma tissues obtained from the Human Protein Atlas (HPA) database. Scale bar: 100 μm.

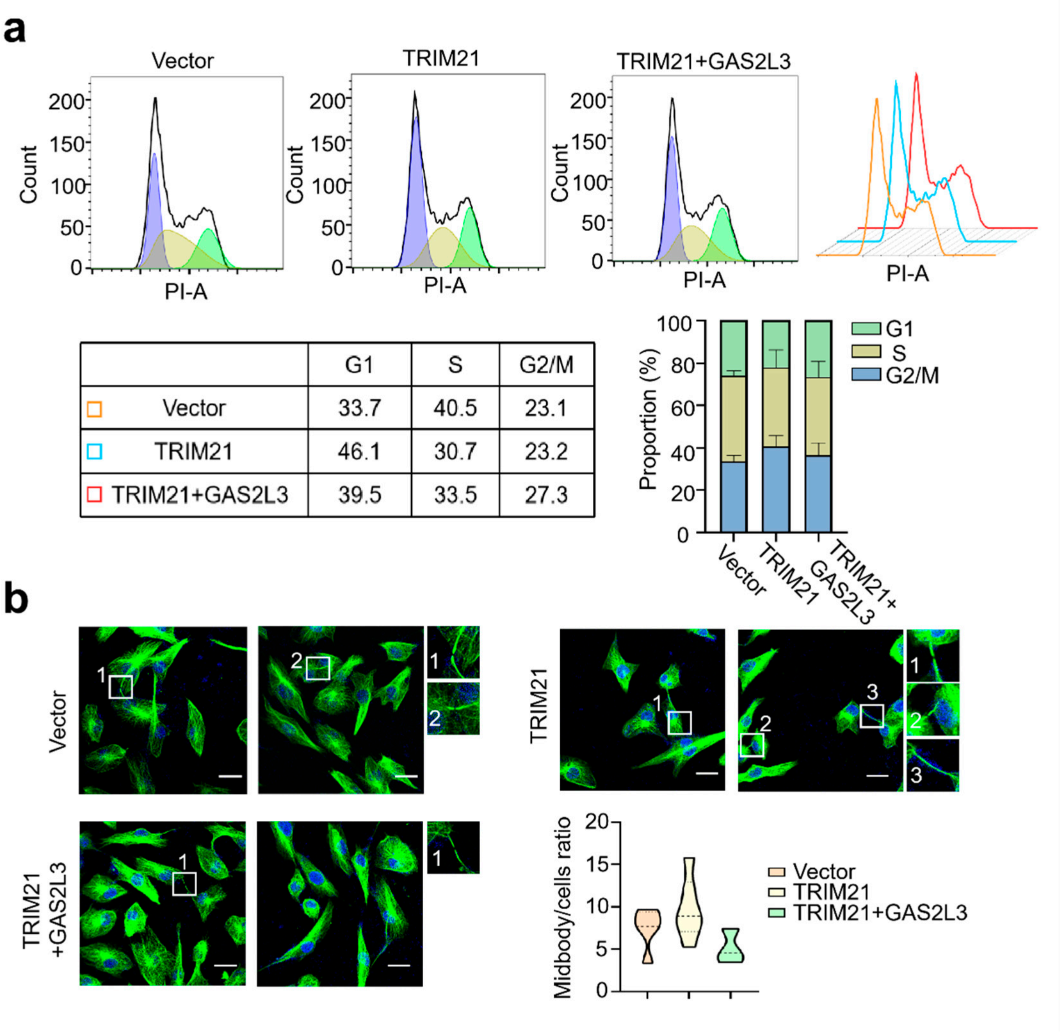


| | G1 | S | G2/M |
|---|---|---|---|
| Vector | 33.7 | 40.5 | 23.1 |
| TRIM21 | 46.1 | 30.7 | 23.2 |
| TRIM21+GAS2L3 | 39.5 | 33.5 | 27.3 |

**Figure S4.** Cell-cycle distribution and midbody frequency analysis in MDA-MB-231 cells. (a) Flow cytometry with PI staining was performed to assess cell-cycle distribution. Representative histograms, tabulated phase fractions and stacked bar graphs showing the proportions of G1, S, and G2/M populations are presented. (b) α-tubulin immunofluorescence was performed to assess midbody abundance during cytokinesis. Representative images with magnified insets of midbody structures are shown, and the midbody/cells ratio was calculated. Scale bar: 10 μm. Statistical analysis was conducted via ordinary one-way ANOVA (ns, $p > 0.05$).
